# Effects of dexmedetomidine nasal spray on perioperative anxiety and quality of postoperative recovery in patients undergoing modified radical mastectomy for breast cancer

**DOI:** 10.3389/fmed.2026.1848029

**Published:** 2026-06-15

**Authors:** Qi Han, Zichao Li, Hongyi Xiao, Fanceng Ji, Chunhui Zheng, Qinglan Li

**Affiliations:** 1 Department of Anesthesiology, Weifang People's Hospital, Weifang, China; 2 Department of Dermatology, Weifang Institute of Dermatology and Venereology, Weifang, China; 3 Department of Breast Diseases, Weifang People's Hospital, Weifang, China

**Keywords:** dexmedetomidine, modified radical mastectomy, nasal spray, perioperative anxiety, quality of postoperative recovery

## Abstract

**Background:**

Dexmedetomidine possesses sedative, analgesic, and anxiolytic properties, but there is still a lack of sufficient clinical data regarding its preoperative intranasal administration. The present study was designed to evaluate whether preoperative intranasal dexmedetomidine spray could alleviate perioperative anxiety and improve postoperative recovery quality in patients with breast cancer undergoing modified radical mastectomy.

**Methods:**

Ninety patients undergoing modified radical mastectomy were randomly assigned to either the dexmedetomidine group (Group D, *n* = 45) or the control group (Group C, *n* = 45). Group D received preoperative intranasal dexmedetomidine spray, whereas Group C received an equal volume of intranasal saline. The primary outcome was the Perioperative Anxiety Scale-7 (PAS-7) score at 30 min after administration. Secondary outcomes included Quality of Recovery-15 (QoR-15) scores at 24 and 48 h after surgery, Asensius Insomnia Scale (AIS) scores during the first and second postoperative nights, perioperative vital signs and BIS values, intraoperative anesthetic consumption, vasoactive drug use, emergence time, and baseline characteristics.

**Results:**

The PAS-7 score at 30 min after administration was significantly lower in Group D than in Group C (9.71 ± 2.07 vs. 12.44 ± 1.98, *P* < 0.001). QoR-15 scores at both 24 and 48 h postoperatively were significantly higher in Group D than in Group C (120.11 ± 6.90 vs. 111.27 ± 7.14, *P* < 0.001; 127.04 ± 6.39 vs. 123.20 ± 6.37, *P* = 0.005). No significant between-group differences were observed in AIS scores, intraoperative hemodynamic variables at most time points, anesthetic consumption, or vasoactive drug use. Time to emergence was significantly longer in Group D than in Group C (8.36 ± 2.79 vs. 6.36 ± 2.83, *P* = 0.001). Baseline characteristics were comparable between the two groups.

**Conclusion:**

Preoperative intranasal dexmedetomidine spray effectively alleviated preoperative anxiety and improved early postoperative quality of recovery in patients undergoing modified radical mastectomy, without significant adverse effects on intraoperative hemodynamics, although time to emergence was prolonged.

## 1 Introduction

Breast cancer incidence has increased steadily in recent years and has remained the most commonly diagnosed malignancy among women, while also representing the second leading cause of cancer-related mortality in this population (1). Surgery is the primary treatment modality for breast cancer. Influenced by female physiological characteristics, social roles, educational level, family financial status, and fertility-related concerns, patients with breast cancer are more likely to experience perioperative anxiety. The reported prevalence of anxiety in this population is as high as 41.9% (2). Perioperative anxiety may induce postoperative cognitive dysfunction and cardiovascular complications, impair postoperative recovery and compliance with subsequent treatment, and ultimately lead to adverse clinical outcomes and long-term behavioral changes (3–6).

A range of strategies has been adopted to improve perioperative anxiety and postoperative recovery, including intensified preoperative education, earplug use, melatonin, and sedative-hypnotic medications (7, 8). Compared with traditional intravenous administration or intranasal delivery via syringe instillation, dexmedetomidine hydrochloride nasal spray has several potential advantages, including non-invasive administration, greater convenience, more precise dosing, and higher bioavailability (9). Previous studies have confirmed its efficacy and safety in gynecologic laparoscopic surgery, bronchoscopy, and otorhinolaryngologic surgery (10–12).

Although intranasal dexmedetomidine has shown potential clinical value, its role in alleviating perioperative anxiety and promoting postoperative recovery in patients undergoing modified radical mastectomy has not been well established. In light of this knowledge gap, we conducted a prospective study to examine the effects of preoperative intranasal dexmedetomidine spray in this surgical population and to explore its potential relevance for perioperative care.

## 2 Materials and methods

### 2.1 Study design and participants

This study was designed as a prospective, randomized, double-blind, single-center clinical trial. Ninety female patients with breast cancer who were scheduled for modified radical mastectomy at Weifang People's Hospital from December 2025 to February 2026 were consecutively recruited. Ethical approval was granted by the Ethics Committee of Weifang People's Hospital (No. KYLL20250930-5), and the trial was prospectively registered in the Chinese Clinical Trial Registry (No. ChiCTR2500114081). All participants provided written informed consent prior to inclusion.

### 2.2 Inclusion and exclusion criteria

The inclusion criteria were as follows: female patients aged 18–65 years scheduled for modified radical mastectomy for breast cancer; body mass index (BMI) of 18.5–27.9 kg/m^2^; and American Society of Anesthesiologists (ASA) physical status I–II.

The exclusion criteria were as follows: long-term medication use for primary sleep disorders; long-term use of antipsychotic drugs; severe chronic sinusitis, nasal deformity, or other significant nasal diseases; and severe bradycardia (heart rate < 50 beats/min) or second-degree or higher atrioventricular block.

### 2.3 Randomization and blinding

Eligible patients were randomly assigned, using a random number table, to either the intranasal dexmedetomidine group (Group D) or the control group (Group C), with 45 patients in each group. After enrollment, the allocation sequence was placed in sealed opaque envelopes. An anesthesia nurse responsible for study drug preparation prepared the investigational medication according to group assignment and then handed it to a blinded anesthesiologist. The anesthesiologist administered either intranasal dexmedetomidine or normal saline. All study data were recorded by another anesthesia nurse who was also blinded to group allocation. The patients, anesthesiologists, and surgeons were all unaware of the treatment assignments throughout the study.

### 2.4 Anesthetic management

Patients were instructed to refrain from solid food for 8 h and from clear liquids for 2 h before surgery. In the pre-anesthesia area, intravenous access was secured and routine parameters, including NIBP, SpO_2_, ECG, and BIS, were monitored. Preoperative anxiety at baseline was assessed by an anesthesia nurse using PAS-7.

In Group D, intranasal dexmedetomidine spray (RA25002, Sichuan Purity Pharmaceutical Co., Ltd.) was administered based on body weight: 3 sprays (75 μg) for patients weighing ≥ 70 kg and 2 sprays (50 μg) for those weighing < 70 kg. In Group C, an equivalent volume of intranasal saline was used instead. Subsequently, all patients received an ultrasound-guided serratus anterior plane block with 20 mL of 0.25% ropivacaine. At 30 min after intranasal administration, patients were brought to the operating room, and PAS-7 was measured again before anesthesia induction.

Once in the operating room, standard intraoperative monitoring was applied. Intravenous dexamethasone 5 mg, flurbiprofen axetil 50 mg, and glycopyrrolate 0.2 mg were given before induction. Anesthesia was induced with sufentanil 0.3 μg/kg, propofol 2 mg/kg, and rocuronium 0.6 mg/kg, followed by tracheal intubation after adequate muscle relaxation. Maintenance of anesthesia was achieved with continuous propofol (6–8 mg/kg/h) and remifentanil (0.1–0.2 μg/kg/min), titrated to maintain BIS between 40 and 60.

When mean arterial pressure (MAP) fell to ≤ 65 mmHg or decreased by more than 30% from the baseline value, intravenous ephedrine 6 mg was given. If the heart rate (HR) dropped below 45 beats/min, atropine 0.3 mg was administered intravenously. At the completion of surgery, infusions of propofol and remifentanil were stopped, and sugammadex 2 mg/kg was given to antagonize residual neuromuscular blockade. Tracheal extubation was carried out after the patient was awake, responded to verbal commands by opening the eyes, resumed spontaneous ventilation, and was able to obey simple instructions. Once vital signs were stable, the patient was transferred to the post-anesthesia care unit (PACU) accompanied by the anesthesiologist and an operating room nurse.

### 2.5 Outcome measures

The primary endpoint was the PAS-7 score measured 30 min after administration of the study drug. Pre- and post-administration anxiety was evaluated using the Preoperative Anxiety Scale-7 (PAS-7), a self-administered instrument developed specifically for Chinese surgical patients. This scale contains seven items addressing both psychological and somatic components of anxiety, with total scores ranging from 0 to 28; higher scores reflect greater anxiety severity.

Secondary endpoints were defined as follows. Postoperative recovery quality was evaluated at 24 and 48 h after surgery with the Quality of Recovery-15 (QoR-15) questionnaire. QoR-15 is a well-validated and reliable tool commonly used to assess postoperative recovery and has demonstrated good clinical utility. It comprises 15 items spanning five domains: pain, physical comfort, physical independence, psychological support, and emotional wellbeing. Total scores range from 0 to 150, with higher values indicating better recovery quality.

Sleep status was assessed on the first and second postoperative nights using the Athens Insomnia Scale (AIS). The AIS consists of eight items, with the first five focusing on nighttime sleep problems and the remaining three evaluating daytime impairment. Each item is rated on a 4-point scale from 0 to 3, resulting in a total score of 0–24. An AIS score of 6 or above was considered indicative of sleep disturbance (13).

Mean arterial pressure (MAP), heart rate (HR), pulse oxygen saturation (SpO_2_), and Bispectral Index (BIS) were measured at prespecified perioperative intervals: before study intervention (T0), immediately prior to anesthesia induction (T1), after tracheal intubation (T2), at skin incision (T3), 30 min and 60 min after the start of surgery (T4 and T5, respectively), at the end of the operation (T6), and 1 min after extubation (T7).

Additional intraoperative data included maintenance consumption of propofol and remifentanil, administration of vasoactive medications, and emergence time, which was defined as the time from cessation of anesthetic agents to eye opening on verbal stimulation. Demographic and clinical baseline data, including age, height, weight, BMI, ASA status, and surgical duration, were likewise recorded.

### 2.6 Sample size estimation and statistical analysis

Statistical processing was conducted with GraphPad Prism, version 10. Prior to formal recruitment, a pilot study involving 15 participants per group was undertaken. In that preliminary analysis, the mean PAS-7 values were 9.71 and 11.35, respectively, and the standard deviation was 2.44. On the basis of these findings, sample size was determined using a two-sided independent-samples *t*-test with an α level of 0.05 and a power of 0.80. The calculation showed that at least 36 patients were required in each group. To account for possible loss to follow-up or withdrawal, the number was increased by 20%, resulting in a final target of 45 patients per group.

Normally distributed continuous data were described as mean ± SD and analyzed with the independent-samples *t*-test. Continuous variables that did not follow a normal distribution were presented as median (IQR) and compared using the Wilcoxon rank-sum test. Categorical variables were expressed as counts and percentages, and group differences were examined with either the chi-square test or Fisher's exact test when appropriate. For repeated observations, two-way repeated-measures ANOVA was applied. Statistical significance was defined as a two-sided *P* < 0.05.

## 3 Results

### 3.1 Participant flow and baseline characteristics

**Figure 1:**
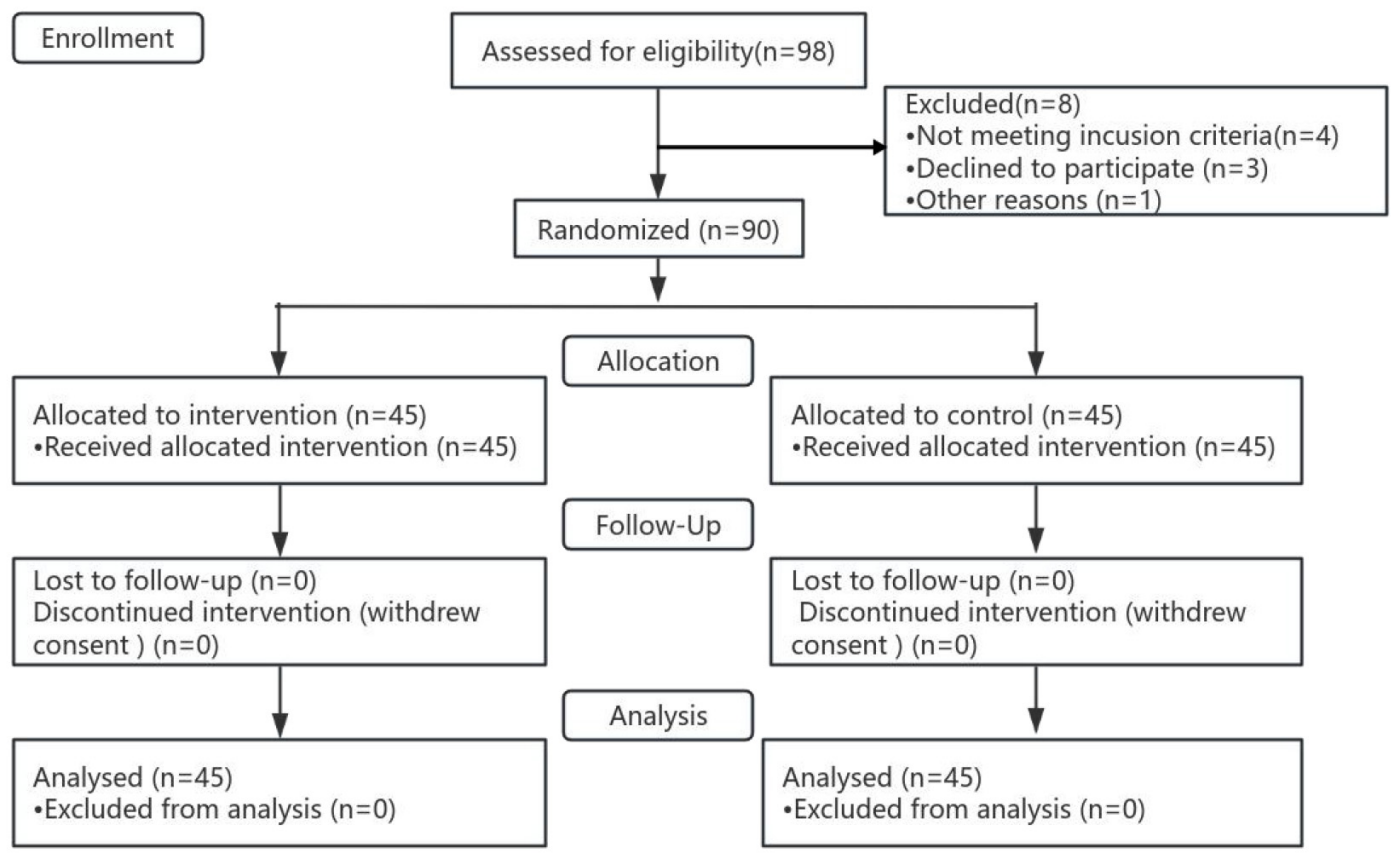
CONSORT flow diagram of patient screening, enrollment, randomization, and analysis.

During the study period, 98 patients scheduled to undergo modified radical mastectomy for breast cancer were screened for eligibility. Eight patients were excluded, including four who did not meet the inclusion criteria, three who declined to participate, and one who was enrolled in another study. The remaining 90 patients were randomly assigned to either Group D or Group C, with 45 patients in each group, and all were included in the final statistical analysis (Figure 1).

No significant differences were observed between the two groups with respect to age, height, body weight, BMI, ASA physical status, or duration of surgery (all *P* > 0.05; Table 1). These results indicated that the baseline characteristics were comparable between the two groups.

**Table 1:** Baseline characteristics of patients in Groups D and C.

Variable	Group D	Group C	t/x^2^	*P*
Age (years)	48.13 ± 10.20	51.16 ± 9.32	−1.467	0.146
Height (cm)	161.11 ± 5.10	159.87 ± 4.57	1.219	0.226
Body weight (kg)	60.91 ± 7.59	61.40 ± 8.29	−0.292	0.771
BMI (kg/m^2^)	23.57 ± 2.81	23.96 ± 2.76	−0.665	0.508
ASA physical status, *n* (%)				
I	20 (44.4%)	17 (37.8%)		
II	25 (55.6%)	28 (62.2%)	0.413	0.520
Duration of surgery (min)	112.20 ± 21.49	106.69 ± 18.47	1.305	0.195

Data are presented as mean ± SD or n (%). Continuous variables were compared using the independent-samples *t*-test, and categorical variables were compared using the χ^2^ test. ASA, American Society of Anesthesiologists; BMI, body mass index.

### 3.2 Comparison of PAS-7 scores before and after administration

There was no significant difference in PAS-7 scores between the two groups before drug administration (12.78 ± 1.76 vs. 12.67 ± 1.76, *P* = 0.765). However, at 30 min after administration, the PAS-7 score was significantly lower in Group D than in Group C (9.71 ± 2.07 vs. 12.44 ± 1.98, *P* < 0.001; Table 2).

**Table 2:** Comparison of PAS-7 scores before and after study drug administration between Groups D and C.

PAS-7 score	Group D	Group C	*t*	*P*
Before administration	12.78 ± 1.76	12.67 ± 1.76	0.300	0.765
30 min after administration	9.71 ± 2.07	12.44 ± 1.98	−6.389	< 0.001

Data are presented as mean ± SD. PAS-7, Preoperative Anxiety Scale-7.

### 3.3 Postoperative quality of recovery and sleep quality

The QoR-15 scores at 24 and 48 h postoperatively were significantly higher in Group D than in Group C (24 h: 120.11 ± 6.90 vs. 111.27 ± 7.14, *P* < 0.001; 48 h: 127.04 ± 6.39 vs. 123.20 ± 6.37, *P* = 0.005), indicating better postoperative quality of recovery in Group D.

No significant differences were found between the two groups in AIS scores on the first postoperative night (4.71 ± 1.94 vs. 5.31 ± 1.72, *P* = 0.124) or the second postoperative night (3.93 ± 1.51 vs. 3.93 ± 1.57, *P* = 1.000). On the first postoperative night, the incidence of sleep disturbance was lower in Group D than in Group C [17/45 (37.8%) vs. 22/45 (48.9%)], although the difference was not statistically significant (*P* = 0.288). On the second postoperative night, the incidence of sleep disturbance decreased in both groups, and no significant between-group difference was observed [9/45 (20.0%) vs. 11/45 (24.4%), *P* = 0.612; Table 3].

**Table 3:** Postoperative quality of recovery and sleep quality in Groups D and C.

Variable	Group D	Group C	*P*
QoR-15 score			
24 h postoperatively	120.11 ± 6.90	111.27 ± 7.14	< 0.001
48 h postoperatively	127.04 ± 6.39	123.20 ± 6.37	0.005
AIS score			
First postoperative night	4.71 ± 1.94	5.31 ± 1.72	0.124
Sleep disturbance, *n* (%)	17 (37.8%)	22 (48.9%)	0.288
No sleep disturbance, *n* (%)	28 (62.2%)	23 (51.1%)	
Second postoperative night	3.93 ± 1.51	3.93 ± 1.57	1.000
Sleep disturbance, *n* (%)	9 (20.0%)	11 (24.4%)	0.612
No sleep disturbance, *n* (%)	36 (80.0%)	34 (75.6%)	

Data are presented as mean ± SD or n (%). QoR-15 and AIS scores were compared using the independent-samples t-test, and the incidence of sleep disturbance was compared using the χ^2^ test. QoR-15, Quality of Recovery-15; AIS, Athens Insomnia Scale.

### 3.4 Hemodynamic variables at different time points

Repeated-measures analysis of variance showed no significant group-by-time interaction for MAP, indicating a similar temporal trend in the two groups (*F* = 1.666, *P* = 0.140). However, MAP at T3 was significantly lower in Group D than in Group C (*P* = 0.015).

For HR, a significant group-by-time interaction was observed (*F* = 3.119, *P* = 0.011). HR was significantly lower in Group D than in Group C at T1, T2, and T3 (*P* = 0.012, *P* = 0.033, and *P* = 0.011, respectively).

**Figure 2:**
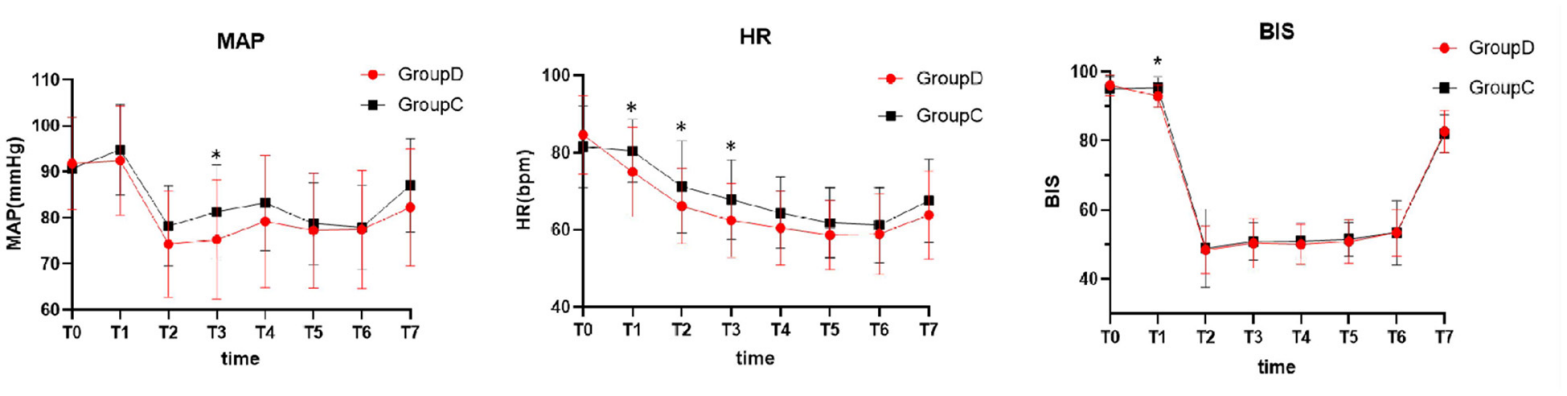
Changes in mean arterial pressure (MAP), heart rate (HR), and bispectral index (BIS) at different perioperative time points in Groups D and C. *P < 0.05 indicates a significant difference between the two groups at the same time point.

No significant group-by-time interaction was found for BIS, suggesting a comparable overall trend between groups (*F* = 0.770, *P* = 0.565). Nevertheless, BIS at T1 was significantly lower in Group D than in Group C (*P* < 0.001; Figure 2).

### 3.5 Intraoperative anesthetic consumption, vasoactive drug use, and emergence time

No significant differences were observed between the two groups in intraoperative propofol consumption (798.77 ± 186.70 mg vs. 805.89 ± 326.91 mg, *P* = 0.899) or remifentanil consumption (0.98 ± 0.18 mg vs. 0.99 ± 0.21 mg, *P* = 0.791). Ephedrine was required in 6 patients (13.3%) in Group D and 4 patients (8.9%) in Group C, with no significant between-group difference (*P* = 0.502).

Compared with Group C, emergence time was significantly prolonged in Group D (8.36 ± 2.79 min vs. 6.36 ± 2.83 min, *P* = 0.001; Table 4).

**Table 4:** Comparison of time to emergence, intraoperative anesthetic consumption, and vasoactive drug use between Groups D and C.

Variable	Group D	Group C	*P*
Time to emergence (min)	8.36 ± 2.79	6.36 ± 2.83	0.001
Propofol (mg)	798.77 ± 186.70	805.89 ± 326.91	0.899
Remifentanil (mg)	0.98 ± 0.18	0.99 ±0.21	0.791
Ephedrine use, *n* (%)	6 (13.3%)	4 (8.9%)	0.502

Data are presented as mean ± SD or *n* (%). Continuous variables were compared using the independent-samples *t*-test, and categorical variables were compared using the χ^2^ test.

## 4 Discussion

This study aimed to evaluate the effects of intranasal dexmedetomidine spray on preoperative anxiety, postoperative quality of recovery, and sleep quality in patients undergoing modified radical mastectomy for breast cancer. Previous studies have shown that intranasal dexmedetomidine spray at doses of 25–200 μg is effective and safe in healthy volunteers (14). In the present study, intranasal dexmedetomidine spray was administered at doses of 50 or 75 μg according to body weight, and the results demonstrated that this regimen significantly reduced PAS-7 scores, effectively alleviated preoperative anxiety, and improved early postoperative quality of recovery, without causing apparent adverse hemodynamic effects. These findings suggest that intranasal dexmedetomidine spray at this dose range may have favorable efficacy and safety in clinical practice.

Perioperative anxiety is common in surgical patients and appears to be particularly prevalent among women (15–17). In patients with breast cancer, perioperative anxiety may be further amplified by concerns related to tumor prognosis, body image, family roles, financial burden, and fertility. Perioperative anxiety is generally characterized by negative emotional responses such as fear, worry, tension, and irritability in response to surgery- and anesthesia-related stress. Although its precise mechanisms remain incompletely understood, previous evidence suggests that excessive activation of the locus coeruleus–noradrenergic (LC-NE) system plays a key role in anxiety under acute stress conditions (18). As a highly selective α2-adrenoceptor agonist, dexmedetomidine exerts anxiolytic effects by inhibiting excessive LC-NE neuronal activation through stimulation of central presynaptic α2 receptors (19). In addition, its anxiolytic effects may also be related to modulation of oxidative stress and suppression of inflammatory mediator release (20, 21). In the present study, PAS-7 scores at 30 min after administration were significantly lower in Group D than in Group C, indicating that preoperative intranasal dexmedetomidine effectively alleviated preoperative anxiety in this patient population. Because PAS-7 incorporates both psychological and somatic dimensions of anxiety and was specifically developed for Chinese surgical patients, it may be particularly suitable for capturing the mixed emotional and physical manifestations of preoperative anxiety in this setting (22).

Traditionally, dexmedetomidine has mainly been administered intravenously or as a self-prepared intranasal instillation, both of which have practical limitations. Intranasal spray represents a flexible and novel route of administration that is non-invasive, convenient, accurately dosed, and associated with high bioavailability, while producing effects comparable to those of intravenous administration (23). In the present study, preoperative intranasal dexmedetomidine spray at 50–75 μg significantly reduced PAS-7 scores 30 min after administration, whereas no obvious change was observed in the control group. These findings suggest that intranasal dexmedetomidine has favorable anxiolytic properties and are consistent with the findings reported by Fan H et al. in lower-limb orthopedic surgery (24). In addition, BIS at T1 was significantly lower in Group D than in Group C, indicating that intranasal dexmedetomidine spray placed patients in a relatively relaxed state characterized by calmness and drowsiness while remaining arousable, with minimal respiratory compromise. This is in line with the known pharmacological action of dexmedetomidine on α2 receptors in the locus coeruleus, through which it induces and maintains a sleep-like state resembling natural non-rapid eye movement sleep, making it particularly suitable for patients with marked preoperative tension or anxiety.

Anesthesia and surgery trigger stress responses and immune activation, resulting in hormonal and inflammatory changes that may adversely affect postoperative recovery. In recent years, both clinical and experimental studies have shown that dexmedetomidine can suppress the release of inflammatory mediators and oxidative stress, as well as attenuate increases in cortisol and C-reactive protein, thereby improving postoperative emotional status and promoting recovery (20, 21). Previous studies have also shown that perioperative intravenous dexmedetomidine can reduce the surgical stress response in elderly patients, decrease inflammatory mediator release, improve postoperative pain, and facilitate recovery (25). In the present study, patients in Group D had significantly higher QoR-15 scores at both 24 and 48 h after surgery than those in Group C, indicating improved early postoperative quality of recovery. Importantly, QoR-15 is a multidimensional outcome that reflects not only pain but also comfort, independence, emotional status, and overall postoperative wellbeing. Therefore, the beneficial effect of intranasal dexmedetomidine spray on QoR-15 may not be explained solely by analgesia, but may instead reflect a broader optimization of the perioperative experience through improved preoperative emotional stability and reduced subjective stress. This interpretation is also consistent with previous studies reporting that dexmedetomidine improves postoperative recovery quality in patients undergoing thoracoscopic surgery (26).

Postoperative sleep disturbance has a substantial impact on postoperative recovery and should not be overlooked. It has been reported that 15% to 72% of patients experience postoperative sleep disturbance after major surgery, and its occurrence is associated with multiple factors, including female sex, preoperative anxiety, age, postoperative pain, postoperative nausea and vomiting, and ward environment (27–29). Some studies have shown that continuous intraoperative infusion of low-dose dexmedetomidine (0.2–0.4 μg·kg^−1^·h^−1^) significantly reduces the incidence of severe sleep disturbance on the day of surgery (30). Other studies, however, have suggested that intraoperative dexmedetomidine infusion does not significantly improve postoperative sleep disturbance overall, but may optimize sleep architecture by reducing nocturnal awakenings and increasing deep sleep, thereby providing indirect benefits for sleep quality (13). With regard to intranasal dexmedetomidine spray, previous evidence has shown that administration at bedtime on the night of surgery can improve postoperative sleep, as reflected by shorter wake time and lower AIS and pain scores (12). In the present study, although the incidence of sleep disturbance on the first and second postoperative nights was numerically lower in Group D than in Group C, the differences were not statistically significant. This finding suggests that preoperative administration of 50–75 μg intranasal dexmedetomidine spray may have limited effects on postoperative sleep in patients undergoing modified radical mastectomy. Several factors may account for this result. First, postoperative sleep is influenced by multiple perioperative and environmental factors beyond preoperative anxiety. Second, a single preoperative dose may not provide sufficient pharmacological coverage for nocturnal sleep after surgery. Third, the sample size of the present study was calculated based on PAS-7 as the primary endpoint and may not have provided adequate statistical power to detect smaller between-group differences in AIS as a secondary outcome.

With regard to safety, the present study showed that preoperative intranasal dexmedetomidine spray at 50–75 μg did not cause clinically meaningful adverse effects on intraoperative hemodynamics. Although MAP, HR, and BIS were lower at certain time points in Group D, no significant difference was observed in the use of vasoactive drugs between groups, suggesting that these changes were generally well tolerated. Compared with intravenous administration, intranasal dexmedetomidine spray may avoid a rapid rise in plasma drug concentration and thereby reduce the risk of respiratory or cardiovascular adverse effects associated with peak concentrations (31). In the present study, emergence time was significantly prolonged in Group D compared with Group C; however, the absolute difference was approximately 2 min. From a clinical perspective, this prolongation may have limited practical impact on the recovery process, although its true relevance should be further evaluated in studies incorporating PACU-related workflow outcomes.

Notably, no significant between-group differences were observed in intraoperative propofol or remifentanil consumption in the present study, which differs from some previous reports (32). One possible explanation is that all patients in this study received preoperative ultrasound-guided serratus anterior plane block, which provided a relatively strong baseline analgesic strategy and may have reduced the overall requirement for intraoperative opioids and anesthetics in both groups. As a result, the potential anesthetic-sparing effect of dexmedetomidine may have been attenuated. In addition, anesthetic depth was titrated to maintain BIS values between 40 and 60, which may have further minimized differences in intraoperative anesthetic consumption between groups.

This study has several limitations. First, this was a single-center study with a relatively limited sample size, and sample size estimation was based on PAS-7 as the primary endpoint; therefore, the statistical power for secondary outcomes such as QoR-15, AIS, and hemodynamic variables may have been insufficient. Second, the study population was limited to female patients aged ≤ 65 years with a BMI between 18.5 and 27.9 kg/m^2^ undergoing modified radical mastectomy for breast cancer; thus, extrapolation of the findings to male patients, older adults, or patients with more complex comorbidities should be made with caution. Third, the present study only evaluated early postoperative recovery quality and short-term sleep outcomes, without assessing longer-term recovery, persistent sleep disturbance, chronic postoperative pain, or adherence to subsequent adjuvant therapy. Finally, anxiety, recovery, and sleep were mainly assessed using subjective questionnaires, and no objective biomarkers or physiological indicators, such as inflammatory mediators, stress hormones, or sleep monitoring parameters, were included. Therefore, mechanistic interpretations should be considered preliminary. Future studies with larger sample sizes, longer follow-up periods, and objective physiological assessments are warranted to further define the optimal role of intranasal dexmedetomidine in perioperative management for breast cancer surgery.

## 5 Conclusion

Preoperative intranasal dexmedetomidine spray significantly alleviated preoperative anxiety and improved early postoperative quality of recovery in patients undergoing modified radical mastectomy for breast cancer, without significant adverse effects on intraoperative hemodynamics, although time to emergence was prolonged.

## Data Availability

The data analyzed in this study is subject to the following licenses/restrictions: Due to hospital ethics, please obtain the raw data from the first author. Requests to access these datasets should be directed to Qi Han, 624326104@qq.com.
